# The role of the gut microbiota-uric acid metabolism axis in high-altitude hyperuricemia: dysregulation mechanisms, pathway associations and therapeutic perspectives

**DOI:** 10.3389/fmicb.2026.1882007

**Published:** 2026-07-21

**Authors:** Chuankai Shi, Lemeng Li, Wenping Ge, Yuan Gao, Yiyang Li, Wuhui Li, Jiangwei Liu

**Affiliations:** 1 Department of Graduate School, Xinjiang Medical University, Ürümqi, China; 2 Key Laboratory of Special Environmental Medicine of Xinjiang, General Hospital of Xinjiang Military Command, Ürümqi, China; 3 The First Clinical Medical College, Xinjiang Medical University, Ürümqi, China

**Keywords:** gut flora, hyperuricemia, hypoxia, intervention strategies, uric acid metabolism

## Abstract

High-altitude areas (≥2500 m) are characterized by low oxygen concentrations, which leads more people affected by high uronic acid in the blood. This review systematically investigates the “gut microbiota-uric acid metabolism axis” as a potential target for inhibiting Hyperuricemia (HUA). This review introduces the four functions of the axis: direct reduction of uric acid, intestinal excretion, regulation of uricase expression, and the intestinal-renal axis signal; then investigates how hypoxia alters all of these paths. In addition, this review illustrates how this axis is related to the classical metabolic pathway of purine synthesis, renal excretion, lactate metabolism, inflammatory-oxidative stress and genetic susceptibility. Adaptation difference: Native highlanders and migrants show different degrees of adjustment to life in the mountains, and migrants are relatively more prone to axis dysfunction. Finally, this review introduces targeted intervention strategies, such as probiotics, prebiotics, fecal microbiota transplantation, and their combination with uric acid-lowering or anti-inflammatory drugs, and put forward a population-stratified precision intervention framework. Overall, this paper provides a theoretical foundation and novel direction for understanding and preventing plateau HUA.

## 1 Introduction

High-altitude environments (≥2500 m) are characterized by low air pressure, hypoxia, intense radiation, and cold climate (Wang et al., 2020). These extreme conditions trigger physiological stress responses, resulting in a higher incidence of plateau-associated diseases than in the plains. Hyperuricemia (HUA) is a chronic metabolic disorder caused by disturbances in purine metabolism or impaired uric acid excretion, which is not only a precursor state of gout, but also closely associated with cardiovascular disease, chronic kidney disease and metabolic syndrome (Sheng et al., 2021). In recent years, the prevalence of HUA has been continuously increasing in Asian countries such as China and South Korea, particularly prominent in high-altitude areas (Yang et al., 2015; Rao and Wang, 2018). Among plains-origin Han Chinese migrants to high-altitude regions, the prevalence of hyperuricemia is substantially elevated, reaching 54.2% in a migrant cohort (Song et al., 2023). The timing of onset is closely related to exposure duration: individuals residing at high altitude for 1–5 years have a 6.7-fold higher risk compared with those staying less than 1 year (Zhu et al., 2025a). Moreover, intrinsic genomic background modulates susceptibility; polymorphisms in urate transporter genes (e.g., *ABCG2*, *SLC2A9*) and hypoxia-adaptive genes (e.g., *HIF1A*) contribute to inter-individual differences in uric acid metabolism and excretion (Zhang et al., 2016; Li et al., 2025). Currently, HUA has become a significant public health issue threatening the health of highland residents and plateau travelers.

The gut microbiota participates in various physiological processes such as nutrient metabolism, immune regulation, and inflammatory responses, which has been identified as an important regulatory factor for systemic uric acid homeostasis (Zhang et al., 2023; Liu et al., 2020). Notably, although the effects of hypoxia on the intestinal barrier and the structure of the microbiota have been gradually clarified (Du et al., 2022a), the specific association between the dysbiosis induced by low-pressure hypoxia and the occurrence of HUA has not yet been systematically reported.

Therefore, this review systematically describes the functional composition of the gut microbiota-uric acid metabolism axis, elucidate the regulatory mechanism of low-pressure hypoxia on this axis, analyze the correlation between the gut microbiota and the classical uric acid metabolism pathway, and compare the phenotypic differences among different populations at high altitudes, aiming to provide a theoretical basis for understanding and preventing plateau HUA.

## 2 Factors influencing plateau hyperuricemia

The development of plateau hyperuricemia is the result of a combination of environmental and biological stresses. The four dimensions of drivers, underlying factors, facilitators and protective factors are described below.

### 2.1 Hypobaric hypoxia

Hypobaric hypoxia is the initiating factor for plateau hyperuricemia, elevating blood uric acid through multiple pathways.

Induction of secondary erythropoiesis. Hypoxia activates the hypoxia-inducible factor pathway, which promotes erythropoietin secretion and stimulates erythropoiesis (Choquenaira-Quispe et al., 2020; Simonson et al., 2010). Erythrocyte renewal accelerates the release of large amounts of nucleic acids, which generate uric acid via the purine metabolic pathway. At the same time, increased blood viscosity and microcirculatory disorders reduce glomerular filtration rate, thereby decreasing uric acid excretion (Du et al., 2022b; Johnson et al., 2013).

Increased uric acid production. Hypoxia inhibits mitochondrial oxidative phosphorylation, ATP synthesis is blocked and catabolism is accelerated. AMP is subsequently metabolized to uric acid precursors such as hypoxanthine and xanthine (Du et al., 2022b). At the same time, the activity of xanthine oxidase, the rate-limiting enzyme of uric acid production, is elevated, accelerating the conversion of substrates to uric acid. A study of a population migrating to an altitude of 4,520 m showed a positive correlation between xanthine oxidase concentration and blood uric acid levels (*P* < 0.001) (Tang, 2016). Intermittent hypoxia also promotes the hydrolytic conversion of xanthine dehydrogenase to xanthine oxidase (Nanduri et al., 2013). In addition, the rs17038412 polymorphism in the xanthine oxidase gene mediates individual differences in susceptibility, with carriers of the AT genotype having a 3.96-fold greater risk of developing the disease than the AA genotype (W, 2015).

Alteration of renal uric acid transporter protein expression. The kidney is the main organ for uric acid excretion, responsible for about 70% of uric acid removal, and the expression level of uric acid transporter proteins directly determines the excretion efficiency (Wen et al., 2024). Hypoxia up-regulates the expression of renal proximal tubular reabsorption transporters urate transporter 1 (URAT1) and glucose transporter 9 (GLUT9), and down-regulates the activity of secretory transporters organic anion transporter 1 (OAT1) and ATP-binding cassette subfamily G member 2 (*ABCG2*), thereby reducing the efficiency of uric acid excretion (Katsurada et al., 2019; Du et al., 2022c; You et al., 2025; Zhu et al., 2025b).

Enhanced lactate competition. Hypoxia can lead to acute kidney injury, when proximal tubular cells undergo metabolic reprogramming toward aerobic glycolysis to maintain reabsorption. The immediate consequence is a significant increase in lactate production (Jiaying et al., 2020). Lactate and uric acid share an organic anion transport system in the kidney; lactate accumulation competitively occupies the transport site and inhibits uric acid secretion (Liu Y. et al., 2024; Yamamoto et al., 1993).

Disruption of intestinal barrier and flora homeostasis. Hypoxia leads to structural abnormalities in the gut, including increased numbers of F4/80 positive cells, swelling of mucus granules and disruption of tight junctions. At the genetic level, hypoxia leads to dysregulated expression of barrier function-related genes involved in antimicrobial activity, immune response, intestinal peristalsis, and nutrient absorption, etc. 16S rDNA sequencing has shown that hypoxia drives intestinal flora dysbiosis, reduces flora abundance and diversity, and in turn disrupts metabolic homeostasis (Wen et al., 2025).

### 2.2 Genetic susceptibility

Genetic variation forms the basis of individual susceptibility to hyperuricemia, acting through multiple genes and pathways.

At the level of uric acid synthesis, genetic variants in XDH, PRPS1, and HPRT1 can enhance the activity of key enzymes of purine metabolism or impair purine recycling, which directly contributes to the overproduction of uric acid (Guo et al., 2022; Nishikura, 2010; Thies et al., 2023). XDH catalyzes the conversion of hypoxanthine to xanthine and xanthine to uric acid. XDH rs17323225 variant may be a genetic risk factor for gout in Mexican populations. PRPS1 mutations result in hyperactive enzyme activity, which is commonly associated with congenital hyperuricemia. HPRT1 defects prevent the recycling of hypoxanthine and guanine into uric acid via XDH, triggering severe hyperuricemia and gout (Yang and Guo, 2022).

At the level of uric acid excretion, *SLC22A12* encodes the major renal uric acid reabsorption transporter URAT1. rs17228129 mutation attenuates the transporter activity, promotes uric acid excretion, and reduces the risk of gout (Yang et al., 2023). rs121907896 and rs121907892 mutations reduce uric acid reabsorption and lower the risk of hyperuricemia (Sakiyama et al., 2016). *SLC2A9* mediates uric acid reabsorption and secretion in renal proximal tubules. Its rs3733591 polymorphism significantly affects blood uric acid levels in the Chinese Han population and Solomon Islanders, and the C allele is associated with gout risk in these populations (Tu et al., 2010). *ABCG2* encodes the uric acid efflux transporter, which is mainly expressed in renal and intestinal epithelial cells. The rs2231142 variant results in decreased transporter function and approximately 40% reduction in renal uric acid excretion, and is the most common global gout risk locus (Dong et al., 2015).

Hypoxia-adapted genes (*HIF1A*, *NOS3*) indirectly affect uric acid metabolism by regulating oxygen metabolism and vascular function. There is a bidirectional regulatory relationship between hyperuricemia and HIF-1α: hypoxia-induced activation of HIF-1α promotes uric acid production, while elevated uric acid in turn up-regulates hepatic HIF-1α expression (Huang et al., 2022), inhibits arginine biosynthesis pathway, and exacerbates hepatic metabolic disorders. In addition, HIF-1α-mediated impairment of vascular endothelial function reduces renal blood perfusion and indirectly decreases the efficiency of uric acid excretion.

Variants in inflammatory and metabolic genes (*NLRP3*, TNF-α, *PPARG*) can amplify the inflammatory response, disrupt metabolic homeostasis, and accelerate disease progression. In addition, environmental factors can regulate gene expression through epigenetic modifications, providing new explanations for environment-gene interactions (Stover et al., 2018).

### 2.3 Lifestyle and co-morbidities

Modifiable risk factors include a diet high in purines, fat and salt, chronic alcohol consumption (especially beer), inadequate water intake, sedentary lifestyle and irregular work and rest. These are the main controllable triggers of morbidity in young and middle-aged groups (Li L. Z. et al., 2023). The risk of gout is significantly higher in developed Western countries than in regions with traditional diets due to high-fat and high-protein dietary patterns (Rai et al., 2017). The Asia-Pacific region has seen a rapid increase in prevalence with dietary westernization. Hypertension, hyperlipidemia, obesity and type 2 diabetes mellitus are bi-directionally associated with hyperuricemia, and gout has become an independent risk factor for cardiovascular disease (Yu et al., 2022). Drugs such as low-dose aspirin, thiazide diuretics and some calcium channel blockers increase the risk of the disease by inhibiting renal uric acid excretion (Tausche et al., 2014). Overlaying the environmental status quo of insufficient health education and low awareness of the disease in some regions, the synergistic effect of multiple factors ultimately contributes to the current global epidemiological characteristics of hyperuricemia and gout, and also puts forward multidimensional and comprehensive requirements for the global comprehensive prevention and control of this disease.

### 2.4 Protective factors

The progression of hyperuricemia and gout is regulated by both risk factors and protective factors. Lifestyle is the most basic exogenous protective factor: high dietary fiber and high vitamin C intake of fruits and vegetables can increase urate solubility, adequate water intake can increase urine output and accelerate uric acid excretion, moderate exercise can improve insulin resistance and reduce the adverse effects of adipokines on uric acid metabolism (C et al., 2011). Klotho protein and intestinal flora are endogenous protective mechanisms, which play a role in the molecular level and micro-ecological level, respectively. Klotho protein is mainly expressed in the renal tubules of kidneys and enhances renal excretion by regulating the activity of uric acid transporter, while reducing renal injury and improving metabolic disorders, and its expression level is negatively correlated with blood uric acid (Lee et al., 2022). Intestinal flora as the regulator of uric acid intestinal excretion, through the production of uric acid enzyme uric acid decomposition into allantoin excretion, supplementation of probiotics, maintenance of intestinal micro-ecological balance can effectively enhance the efficiency of intestinal uric acid metabolism (Wang J. et al., 2022; Wang S. F. et al., 2022).

**FIGURE 1:**
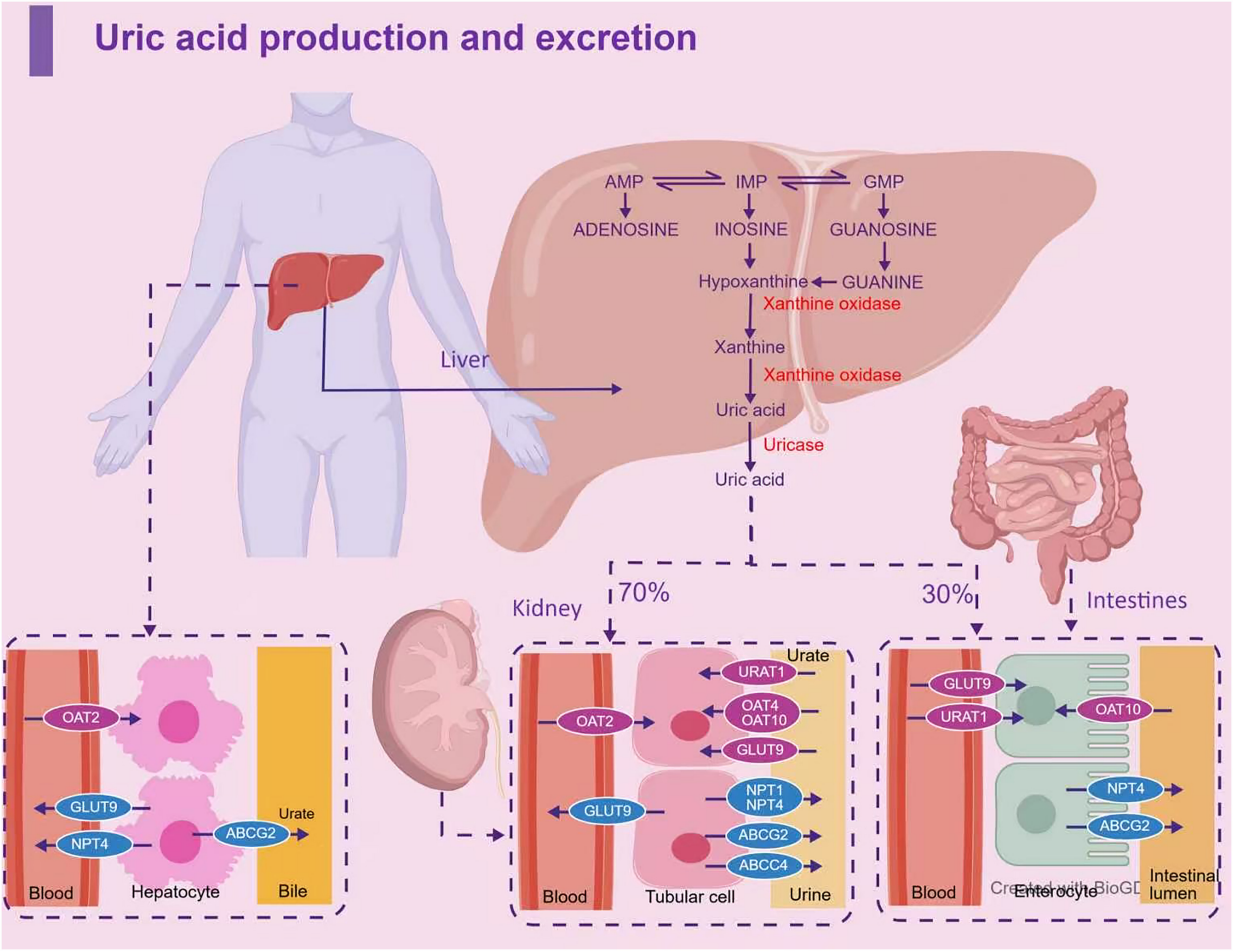
Schematic diagram of classical uric acid metabolism. Uric acid is primarily produced from purine catabolism in the liver (via *XDH*/XOD). Renal excretion accounts for ∼70% of urate elimination, involving glomerular filtration, tubular reabsorption (URAT1, GLUT9), and secretion (*ABCG2*, OAT1). Intestinal excretion (∼30%) is mediated by *ABCG2* and gut bacterial degradation.

As shown in Figure 1, uric acid metabolism mainly includes *de novo* synthesis in the liver, and excretion via the kidney and intestine. This classical pathway provides a fundamental basis for understanding the dysregulation of uric acid homeostasis in high-altitude hyperuricemia.

## 3 The gut flora-uric acid metabolism axis

The term “intestinal flora-uric acid metabolism axis” refers to the systematic uric acid metabolism regulatory network formed by the intestinal flora through the following four pathways: direct degradation of uric acid, regulation of intestinal uric acid excretion, regulation of uric acid synthase activity, and mediation of intestinal-renal axis signaling. The four main functions of this axis are described below.

### 3.1 Direct degradation of uric acid

Intestinal flora is the only exogenous uric acid degradation pathway in the body. *Lactobacillus* spp., *Pseudomonas* spp., and other commensal bacteria secrete uricase, allantoinase, and allantoinase, which progressively degrade uric acid into soluble allantoin, allantoic acid, and even urea, which are excreted via the feces, and directly reduce the systemic uric acid load (Shirvani-Rad et al., 2023; Yin et al., 2022). Clinical studies have shown that the relative abundance of *Prevotella* spp. and *Mycobacterium* spp. in the gut of gout patients is elevated, and the abundance of Enterobacteriaceae is decreased, suggesting that impaired uric acid degradation leads to uric acid accumulation. Supplementation with specific probiotics can partially restore uric acid degradation in these patients (Tong et al., 2022).

### 3.2 Regulation of intestinal uric acid excretion

Short-chain fatty acids produced by the fermentation of butyrate-producing commensal *Clostridia* (e.g., *Clostridium butyricum* and related species) can activate intestinal epithelial signaling pathways, upregulate the expression and activity of the uric acid transporters *ABCG2* and *SLC2A9*, and enhance the secretion of uric acid from the bloodstream to the intestinal lumen (Cui et al., 2026; Matsubayashi et al., 2020). These butyrate-producing bacteria was found to reduce uric acid levels (Shirvani-Rad et al., 2023).

### 3.3 Regulation of uric acid synthase activity

Beneficial bacteria inhibit hepatic and intestinal *XDH*/xanthine oxidase (XOD) activity and reduce the degradation of endogenous purines to uric acid through the production of metabolites or direct interactions. *Lactobacillus fermentum* and *Lactobacillus shortum* produce specific metabolites that reduce XOD activity in liver and intestinal tissues and inhibit uric acid production at source (Rao et al., 2024; Wu et al., 2021). On the contrary, conditionally pathogenic bacteria such as *E. coli* and *Aspergillus* can secrete *XDH* homologous proteins to promote purine degradation and uric acid production (Liu et al., 2023).

### 3.4 Intestinal-renal axis signaling

Intestinal flora modulates intestinal-renal axis function by maintaining intestinal barrier integrity. When the flora is balanced, short-chain fatty acids promote intestinal epithelial tight junction protein synthesis, increase ZO-1 and Occludin expression, inhibit intestinal permeability, and enhance mechanical barrier function. Short-chain fatty acids that enter the blood circulation simultaneously protect renal function and promote renal uric acid excretion (Li et al., 2020). In the case of dysbiosis (e.g., elevated abundance of *Mycobacterium* spp. and *Aspergillus* spp.), overcolonization of pathogenic bacteria damages the intestinal mucosal barrier, leading to leakage of toxins, such as lipopolysaccharides, into the bloodstream and triggering metabolic endotoxemia (Li L. et al., 2023). This activates a systemic inflammatory response that both exacerbates renal injury and reduces the efficiency of uric acid renal excretion, and amplifies gouty inflammation through inflammatory factors, creating a self-perpetuating cycle of dysbiosis, barrier damage, increased inflammation, and uric acid retention (Jhang et al., 2015).

### 3.5 Effect of hypobaric hypoxia on the gut flora-uric acid axis

**FIGURE 2:**
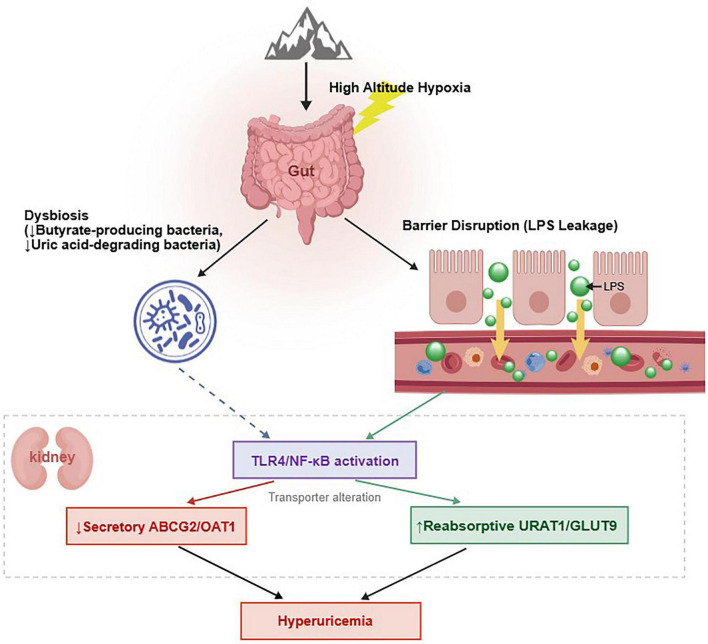
Schematic diagram of the gut microbiota–uric acid axis under hypobaric hypoxia. Hypoxia induces gut dysbiosis (reduced butyrate–producing and uric acid–degrading bacteria) and intestinal barrier disruption, leading to lipopolysaccharide (LPS) leakage into the bloodstream. LPS activates renal TLR4/NF–κB signaling, which downregulates uric acid secretory transporters (*ABCG2*, OAT1) and upregulates reabsorptive transporters (URAT1, GLUT9). Consequently, both intestinal and renal uric acid excretion are impaired, resulting in hyperuricemia.

Low-pressure hypoxia is an important trigger for hyperuricemia and gout in plateau. The prevalence of hyperuricemia in Tibet at an altitude of more than 4,500 m reached 19.66%, which was significantly higher than that of 13.3% in the plains, and the incidence of gout tended to increase with the increase in altitude and the prolongation of years of residence (Xiong and Zhang, 2024). The mechanisms include: hypoxia remodeling of the intestinal flora structure (Li et al., 2016), resulting in a decrease in the abundance of uric acid-degrading bacteria, the increase of conditionally pathogenic bacteria, not only to weaken the exogenous uric acid degradation ability, but also to promote the endogenous purine metabolism and uric acid production; hypoxia and bacterial dysbiosis to damage the intestinal barrier, lipopolysaccharide translocation triggered systemic inflammation, inhibition of renal and intestinal uric acid transporter activity, exacerbating uric acid retention; short-chain fatty acids to reduce the impairment of short-chain fatty acid generation intestinal-renal axis coordination, unable to compensate for the increased endogenous purine load brought about by plateau erythrocytosis, and ultimately driving the elevation of blood uric acid (Yan et al., 2023). Mechanistically, hypobaric hypoxia stabilizes hypoxia-inducible factor-1α (HIF-1α), which bidirectionally interacts with uric acid metabolism to exacerbate uric acid overproduction (Huang et al., 2022). Prolonged hypoxia disrupts intestinal tight junctions and reduces short-chain fatty acid-producing commensal bacteria, leading to lipopolysaccharide translocation into the circulation (Li et al., 2020). Circulating lipopolysaccharide activates the renal TLR4/NF-κB inflammatory pathway, which downregulates the expression of renal and intestinal uric acid transporters (e.g., URAT1, GLUT9, *ABCG2*) and impairs both renal and intestinal uric acid excretion (Bian et al., 2020; You et al., 2025). Reduced short-chain fatty acid production further compromises intestinal *ABCG2*-mediated uric acid secretion (Cui et al., 2026). Collectively, this hypoxia-triggered cascade-dysbiosis-barrier disruption-endotoxemia-transporter downregulation-establishes a causal link from gut-renal axis dysfunction to hyperuricemia under hypobaric hypoxia. The key pathways of the gut microbiota-uric acid axis under hypoxia are summarized in Figure 2.

## 4 Interactions with classical metabolic pathways

The gut flora-uric acid axis does not operate independently, but rather there are interactions with purine synthesis, renal excretion, lactate metabolism, inflammatory-oxidative stress, and genetic susceptibility pathways that collectively influence uric acid homeostasis and hyperuricemia risk.

### 4.1 Purine synthesis

Intestinal flora influence purine metabolism by modulating substrate availability and key enzyme activities. Beneficial bacteria can directly uptake exogenous dietary purines, reduce translocation to the liver, and decrease the substrate load for purine synthesis from scratch (Kano et al., 2018; Yamada et al., 2016). Short-chain fatty acids produced by colony metabolism inhibit hepatic *XDH*/XOD activity and reduce endogenous uric acid production (Deng et al., 2024). During dysbiosis, *Escherichia* spp. and *Shigella* spp. secrete xanthine dehydrogenase, which directly converts hypoxanthine and xanthine to uric acid, elevating blood uric acid levels (Singh et al., 2024). Clinical studies have confirmed that the abundance of purine metabolism-related microbial genes is significantly abnormal in the intestinal flora of patients with gout (Guo et al., 2016), and there is a correlation with blood uric acid levels.

### 4.2 Renal uric acid excretion

About one-third of uric acid is excreted via the intestine. When renal function is impaired, intestinal flora can enhance intestinal excretion by up-regulating ABCG2 and SLC2A9 expression to compensate for inadequate renal excretion (Ohashi et al., 2023). Short-chain fatty acids produced by fermentation of commensal bacteria activate cAMP and ERK1/2 signaling pathways via GPR41/43 receptors, inhibit the release of renal inflammatory factors, and at the same time improve renal perfusion through serotonin-mediated modulation of vascular tone to provide physiological bases for the normal functioning of uric acid transporter proteins and indirectly promote renal uric acid excretion (Foresto-Neto et al., 2021). When the intestinal barrier is damaged by dysbiosis, translocated lipopolysaccharide activates the renal TLR4/NF-κB inflammatory pathway (Bian et al., 2020), which impairs renal function and down-regulates the expression of transporters, exacerbating uric acid retention. This intestinal-renal axis regulatory mechanism constitutes a double guarantee for uric acid excretion (Ren S. Q. et al., 2023).

### 4.3 Inflammatory and oxidative stress pathways

Dysbiosis and barrier damage cause lipopolysaccharide entry into the bloodstream, activate NLRP3 inflammatory vesicles, and promote the release of pro-inflammatory factors such as IL-1β and TNF-α, while exacerbating oxidative stress (Zhang et al., 2025). Urate crystal deposition triggers local inflammation, further disrupting flora homeostasis, reducing the abundance of butyrate-producing beneficial bacteria, and decreasing anti-inflammatory butyrate production. Together, dysbiosis and inflammation weaken the intestinal mucosal barrier, increase permeability, and promote the translocation of harmful substances such as lipopolysaccharide into the bloodstream. These changes upregulate XOD activity, exacerbate the inflammatory response with elevated blood uric acid, and ultimately form a feedback loop involving urate crystal deposition, inflammatory activation, dysbiosis, barrier impairment, and uric acid metabolism disruption, driving gout progression (Jun and Xiao-Fei, 2022). Macrogenomic studies have shown that the gene abundance and α-diversity of intestinal flora in gout patients are significantly reduced, and the abundance of butyric acid-producing bacteria is significantly down-regulated, which is negatively correlated with the elevated serum CRP levels, verifying the close association between intestinal flora and inflammatory response (Chu et al., 2021).

### 4.4 Host genetic background

At the genetic level, there were significant polymorphic differences in uric acid transport-related genes between the plateau-dwelling and plains populations. *ABCG2* rs12505410 was a gout-protective locus in Tibetans, while it was a risk locus in Han Chinese; *SLC2A9* rs7663032 and rs10022499 were associated with a reduced risk of gout in Han Chinese, with no significant association in Tibetans (Zhang et al., 2016). Among them, *ABCG2* rs2231142 (Q141K) is a key genetic variant for gout, which can lead to congenitally impaired intestinal uric acid excretion, and *ABCG2*-mediated decrease in uric acid excretion by about 50%, significantly elevating blood uric acid levels. In both European and Polynesian populations, this variant was strongly associated with gout susceptibility (combined OR = 1.85), and the effect on gout was stronger than that of uric acid regulatory genes such as *SLC2A9* (Wrigley et al., 2020). Protective gene variants also exist in the Tibetan population, and the CC genotype of *SLC22A12* rs559946 was shown to be a protective factor against hyperuricemia (Li et al., 2025). At the flora level, Mendelian randomization studies have confirmed a causal association between intestinal flora and gout (Liu X. et al., 2024), and significant overexpression of purine metabolism-related genes in the intestinal flora of patients with hyperuricemia and gout suggests that altered metabolic function of the flora is involved in the development of the disease (Martínez-Nava et al., 2025). At the interaction level, individuals carrying susceptibility gene variants are inherently deficient in intestinal uric acid excretion, and are more likely to have altered flora homeostasis when exposed to low-pressure hypoxia and high-purine diets, which significantly increases the risk of disease onset, while individuals carrying protective gene variants may be able to resist the negative effects of the environment to a certain extent. This gene-colony-environment association constitutes an important mechanism for the development of plateau hyperuricemia.

## 5 Population differences

### 5.1 Plateau-dwelling populations

Tibetans and other plateau-dwelling populations have developed a gut flora-uric acid axis state adapted to the low-oxygen environment through long-term natural selection (Lv et al., 2023). Globally, the prevalence of hyperuricemia in high altitude-dwelling populations is significantly lower than that in migrants at the same altitude and in high plains populations. The age-standardized prevalence of hyperuricemia in Tibetans living on the Tibetan Plateau (2.05%) is lower than that in mainland China as a whole (6.2%). Gao et al. demonstrated that the abundance of uric acid-degrading microorganisms, such as *Trichoderma* sp., in the intestines of long-term plateau-dwelling populations was significantly higher than that of plains populations, and was correlated with lower levels of uric acid in the intestinal tract (Su et al., 2024). The presence of specific variants in genes such as *ABCG2* and *SLC2A9* was associated with the formation of synergistic fitness in the intestinal flora to form synergistic adaptations. The traditional diet of plateau-dwelling populations (e.g., traditional Tibetan fermented yak milk) naturally contains probiotics that are highly efficient at degrading purines, which helps to alleviate the uric acid load (Wang X. et al., 2025). The result is a cycle of flora homeostasis, metabolic fitness and uric acid balance.

### 5.2 Migrating populations

Due to the lack of long-term environmental adaptation and the corresponding genetic basis, the intestinal flora-uric acid axis of the plains population migrating to the plateau is susceptible to alteration by low oxygen exposure, dietary shifts, and other factors, and the risk of hyperuricemia and gout is significantly elevated. Song et al.’s (2023) survey of 7,070 highland transplants showed that the overall prevalence of hyperuricemia in the migrant population was 54.2% (59.9% in males and 22.5% in females). The plateau-plain comparison study by Wang Q. et al. (2025) confirmed that the prevalence of hyperuricemia in the plateau region (56.43%) was 10.5 times higher than that in the lower altitude region (5.38%) (*P* < 0.001). The Tibetan-Chinese comparative study showed that the prevalence of hyperuricemia among Han Chinese college students who moved to the plateau (28.1%) was significantly higher than that of native Tibetan college students (10.0%), and that Han Chinese ethnicity was an independent risk factor for plateau hyperuricemia (*P* = 0.018) (An et al., 2025a).

In a study of Han Chinese male migrants, Zhu et al. (2025a) found that the duration of plateau exposure was significantly associated with hyperuricemia (OR = 6.744), and the risk of the disease was 6.7 times higher in those exposed for 1–5 years than in those exposed for less than a year. Ma et al. (2025) found significant changes in the flora structure after 7 days of plateau exposure, in a longitudinal study of 406 healthy adult males who had been exposed from a low altitude (800 m) to a high altitude (4,500 m). In a longitudinal study of 406 healthy adult males who had been rushed from low altitude (800 m) to high altitude (4,500 m), Ma et al. (2025) found that significant flora structural changes occurred after 7 days of plateau exposure: an increase in the abundance of opportunistic pathogens and a significant decrease in the number of short-chain fatty acid-producing beneficial bacteria, and that these changes persisted even after returning to low altitude, suggesting that high altitude exposure has a long term remodeling effect on the gut flora. Metabolic disturbances were further exacerbated by alcohol consumption as an independent risk factor for plateau hyperuricemia (*P* < 0.001) (An et al., 2025b). Significant biochemical differences between Tibetans and Chinese form the genetic and metabolic basis for the different etiologies of hyperuricemia in the two regions (Ren X. W. et al., 2023). Migration to the plateau also significantly reshapes gut flora and metabolite profiles, thereby increasing susceptibility to plateau-related health problems (Zhou et al., 2025).

Therefore, unlike the flora homeostasis, metabolic fitness and uric acid homeostatic cycle of the world-dwelling population, the transplanted population presents a pattern of environmental shock, flora dysbiosis and metabolic disorders, making them a high-risk group for plateau hyperuricemia and gout.

## 6 Therapeutic strategies

Modulation of the gut microbiome is a promising strategy for managing hyperuricemia (Yang et al., 2025).

### 6.1 Probiotic supplementation

Probiotics that regulate uric acid metabolism are mainly found in the genera *Lactobacillus*, *Streptococcus*, *Bacillus*, *Schizococcus*, *Bifidobacterium* and *Escherichia*. A variety of *Lactobacillus* strains can directly degrade uric acid, degrade purine nucleosides, inhibit XOD activity, regulate intestinal flora, and reduce blood uric acid (Cheng et al., 2025). For example, *Lactobacillus fermentum* JL-3, isolated from the traditional Chinese fermented food “syrupy water,” can significantly reduce the blood uric acid level of hyperuricemic mice by 31.3% (Wu et al., 2021). *Lactobacillus shortcombicus* DM9218 improved fructose-induced hyperuricemia by degrading inosine and inhibiting hepatic XOD activity (Deng et al., 2017). *Lactobacillus gattii* PA-3 can effectively reduce blood uric acid levels in patients with borderline hyperuricemia (Kamatani et al., 2016). In addition, other genera also have the potential to lower uric acid: *Streptococcus* spp. can degrade hypoxanthine and inosine, reduce blood uric acid and elevate short-chain fatty acids in rats; *Bacillus* spp. can regulate purine metabolism, reduce blood uric acid, and increase the number of butyric acid-producing bacteria; *Schizosaccharomyces papyrus* spp. can degrade uric acid and oxalic acid, and reduce the accumulation of purine nucleosides; *Bifidobacterium* spp. abundance is negatively correlated with blood uric acid; *Bifidobacterium shortum* and *Bifidobacterium longum* can reduce purine accumulation *Bifidobacterium shortum* and *Bifidobacterium longum* can reduce purine accumulation and inflammatory response; *Escherichia* spp. can absorb purine and directly degrade uric acid, and its efficacy is comparable to that of allopurinol (Cheng et al., 2025).

### 6.2 Probiotic supplementation

Prebiotics are non-digestible dietary fibers that selectively stimulate the growth and activity of specific flora by promoting the synthesis of beneficial metabolites. Prebiotics that regulate uric acid metabolism mainly include polysaccharides, polyphenols, bioactive peptides and natural organic acids (Cheng et al., 2025). Polysaccharides play a role by inhibiting XOD/ADA activity, regulating uric acid transport proteins, remodeling intestinal flora, and promoting uric acid excretion. Guo et al. (2021) demonstrated that inulin supplementation increased short-chain fatty acid production, up-regulated ABCG2 expression, inhibited hepatic XOD activity, and repaired the intestinal barrier. Polyphenols inhibit XOD/ADA activity and upregulate OAT1; bioactive peptides inhibit XOD/ADA and regulate transport proteins; and natural organic acids inhibit XOD and upregulate ABCG2 to promote intestinal uric acid excretion (Cheng et al., 2025).

### 6.3 Fecal microbiota transplantation

For refractory gout patients with severe dysbiosis, fecal bacteria transplantation can rapidly rebuild intestinal homeostasis by transplanting healthy donor flora. Clinical studies have confirmed its remarkable efficacy: He Xingxiang’s team treated 11 cases of acute recurrent gout patients with washed microbiota transplantation, the patients’ blood uric acid was significantly reduced, the frequency and duration of gouty attacks were significantly reduced within 1 year, the intestinal barrier function was restored to a healthy level, and only two cases showed mild self-limiting adverse reactions (Xie et al., 2022). A randomized controlled trial of 102 patients with refractory gout by Shuting et al. (2025) showed that the total control rate in the fecal bacteria transplantation group (89.13%) was significantly higher than that in the drug control group (69.57%), with significant reductions in blood uric acid and inflammatory indexes, and elevated abundance of intestinal bifidobacteria and lactobacilli. Animal experiments have likewise confirmed that fecal bacteria transplantation can reduce blood uric acid and XOD activity and restore the balance of intestinal flora in hyperuricemic mice (Yuan et al., 2025). Fecal bacteria transplantation provides a safe and effective treatment option for refractory gout by correcting flora disorders, lowering uric acid, reducing inflammation, and repairing the intestinal barrier.

### 6.4 Combination therapies

Uric acid lowering drugs combined with probiotics/prebiotics. XOD inhibitors such as allopurinol and febuxostat can block uric acid production from the source, but long-term use may cause intestinal flora disorders. Clinical studies have confirmed that after treatment with allopurinol combined with synbiotics, patients’ intestinal flora parameters were stabilized, the reduction of blood uric acid (18.7%) was better than that of the allopurinol alone group (13.3%), and the rate of normalization of uric acid was also higher (40.3% vs. 21%, *P* < 0.01) (Kondratiuk et al., 2020). The rationale is that urate-lowering drugs (e.g., xanthine oxidase inhibitors) reduce endogenous uric acid production, while probiotics/prebiotics enhance intestinal uric acid degradation and excretion through complementary pathways (direct degradation, *ABCG2* upregulation, and barrier protection), thereby achieving additive or synergistic effects. The combination of prebiotics and uric acid-lowering drugs can regulate intestinal flora and improve uric acid metabolism by increasing short-chain fatty acid production (Ren et al., 2025).

Anti-inflammatory drugs combined with probiotics. Colchicine is often required to control inflammation during acute attacks of gout, but its clinical application is limited by intestinal toxicity. Animal experiments have shown that the combination of *Lactobacillus paracasei* GY-1 and colchicine can attenuate colchicine-induced adverse effects such as weight loss, colon shortening, and intestinal barrier damage, as well as enhance its anti-inflammatory effect and restore colchicine-induced imbalance of intestinal flora (Zeng et al., 2024). The rationale for this combination is that colchicine suppresses acute gouty inflammation but damages the intestinal barrier and microbiota; probiotics restore barrier integrity and microbial diversity, counteracting colchicine’s adverse effects while preserving its anti-inflammatory efficacy. The combined intervention strategy provides an optimized solution for the comprehensive management of hyperuricemia and gout by complementing the mechanisms and enhancing the efficacy while mitigating the negative effects of the drugs on the intestinal microecology.

### 6.5 Precision interventions

Individualized stratified intervention strategies based on population characteristics can significantly enhance the effectiveness and safety of treatment.

The traditional diet of world-dwelling populations is high in purines, but the intestinal flora has co-evolved with high purines. Tibetans and other world-dwellers have a high abundance of uric acid-degrading bacteria such as Trichosporonaceae, which can effectively break down purines and uric acid in the intestinal tract. However, modern diets of refined sugar, alcohol, and low-fiber processed foods have destroyed the original flora structure. Therefore, screening different sources of lactic acid bacteria from local artisanal fermented dairy products and making full use of local lactic acid bacteria resources for the treatment of hyperuricemia are new directions (Wang X. et al., 2025). Conventional uric acid-lowering western drugs are selected according to clinical needs.

Interventions in migrant populations should prioritize the re-establishment of intestinal flora with supplementation of multi-strain probiotics combined with prebiotics to repair the intestinal barrier and reduce the inhibition of uric acid excretion by endotoxin entry into the bloodstream. Alcohol and fructose drinks should also be strictly limited.

Patients with comorbidities are often accompanied by a decrease in intestinal butyric acid-producing bacteria, leading to an impaired intestinal barrier and chronic low-grade inflammation that further inhibits uric acid excretion (Attaye et al., 2025). Intervention focuses on supplementation of butyric acid-producing bacteria, which can be achieved by oral *Clostridium butyricum* preparations or increased dietary fiber, and butyric acid improves insulin resistance and attenuates the inflammatory response induced by uric acid crystals.

## 7 Conclusion

In summary, the intestinal flora-uric acid metabolism axis is an important regulatory link in the development of plateau hyperuricemia. In-depth analysis of its molecular mechanism, differences in population adaptation and intervention strategies is of great significance for revealing the pathogenesis of plateau hyperuricemia and formulating precise preventive and control measures.

## 8 Limitations and future perspectives

Several limitations and future directions should be acknowledged. First, the specific molecular mechanisms by which hypoxia regulates the gut flora-uric acid axis remain unclear; multi-omics approaches are needed to elucidate the dynamic regulatory network. Second, most population studies are cross-sectional; longitudinal cohorts on the Tibetan Plateau are warranted to clarify causal relationships among environment, genetics, flora, and hyperuricemia. Third, the comparison between native highlanders and migrants remains superficial; future studies should systematically integrate genetic background, gut microbiota composition, dietary patterns, and hypoxic adaptation using unified altitude stratification and duration-response analysis to disentangle their interactions. Fourth, randomized controlled trials validating probiotic and fecal transplantation efficacy in plateau environments are still lacking. Fifth, most intervention evidence originates from non-high-altitude studies or animal models; direct high-altitude human trials are scarce, and no formal evidence grading has been applied. Therefore, the translational potential should be interpreted with caution. Developing plateau-adapted microecological agents and their combination with uric acid-lowering drugs represents a promising translational direction.
